# Multiple Tubercular Intestinal Perforations: A Case Report

**DOI:** 10.7759/cureus.66352

**Published:** 2024-08-07

**Authors:** Varun Shetty, Mathew John Mathai, Iqbal M Ali

**Affiliations:** 1 General Surgery, Dr. D. Y. Patil Medical College Hospital and Research Center, Dr. D. Y. Patil Vidyapeeth (Deemed to be University) Pimpri, Pune, IND

**Keywords:** pneumoperitoneum, small bowel perforation, tuberculosis, non-bilious vomiting, abdominal pain

## Abstract

Intestinal tuberculosis (TB) is a frequently encountered pathology by surgeons all over India. There exists a vast body of knowledge about this disease; however, a detailed understanding of its presentation as well as surgical management is essential for every Indian surgeon, given its rampant nature. This report discusses the case of a 28-year-old female presenting with severe left upper abdominal pain, non-bilious vomiting, and fever, who was ultimately diagnosed with small bowel TB leading to perforations.

Despite a history of pulmonary TB treated a year prior, the patient exhibited significant clinical and imaging findings, including pneumoperitoneum and peritonitis. Exploratory laparotomy revealed multiple tubercular perforations in the mid-jejunum and a stricture causing proximal jejunal dilatation. Surgical intervention involved resection of the affected segment and end-to-end anastomosis. Histopathological analysis confirmed TB as the cause. This case underscores the importance of considering TB in the differential diagnosis of small bowel perforations and highlights the critical role of timely surgical intervention and comprehensive management in improving patient outcomes.

## Introduction

A hollow viscus perforation and the resulting peritonitis are a relatively common surgical emergency that requires exploration and repair. The majority of perforations that occur on a daily basis are caused by peptic ulcers. Although hollow viscus perforations are uncommon, small bowel and colonic perforations contribute to the caseload [1,2]. Trauma and enteric fever are the most common causes of small intestine perforations, but non-specific ileal perforations can also occur [3]. Even in areas where tuberculosis (TB) is prevalent, such as India, small intestinal perforation remains a rare complication of the disease. Extrapulmonary TB accounts for approximately 12% of all TB cases, with intestinal TB making up 11-16% of the total. It thus represents 1-3% of all TB cases [4]. Although the government has made significant efforts to control the disease, and despite the availability of effective anti-tubercular medications, it remains a major cause of morbidity and mortality in our country. The incidence of intestinal TB was approximately 1.3 per 100 admitted patients as per one study [5].

The diagnosis of abdominal TB remains a persistent challenge, particularly for surgeons. The subtle clinical presentation and proclivity to resemble a variety of other pathologies, such as Crohn's disease and sarcoidosis, all contribute to the difficulty and delay in diagnosis [5]. The misdiagnosis rate can range from 50 to 70%. Furthermore, some Crohn's disease patients have been reported to have positive AFB staining, which adds to the confusion. However, a thorough clinical examination followed by a definitive endoscopy can help the surgeon make the correct diagnosis. The key to reducing the morbidity and mortality associated with intestinal TB is timely diagnosis and appropriate treatment. The administration of anti-tubercular medication per the Revised National Tuberculosis Control Programme (RNTCP) is central to the management of intestinal TB. Isoniazid, rifampicin, pyrazinamide, and ethambutol are the most commonly used drugs. However, the rising incidence of multi-drug resistant TB (MDR-TB) necessitates individualized treatment with second-line drugs [6].

Although approximately 70% of cases of intestinal TB can be treated medically, a few cases develop complications such as intestinal obstruction, perforation, stricture formation, peritonitis, and so on. These complications necessitate immediate surgical intervention (resection and anastomosis/diversion procedure) to reduce associated morbidity and mortality [7]. We present a case of a patient with multiple tubercular ileal perforations that resulted in peritonitis and was treated at our institution.

## Case presentation

A 28-year-old female presented to the surgical outpatient department of a tertiary health center with a seven-day history of left upper abdominal pain and non-bilious vomiting over the past three days. She denied any symptoms of melena, hematochezia, or hematemesis. Additionally, she reported a fever with chills for the last three days. Her medical history included a diagnosis of pulmonary TB two years prior, for which she had completed nine months of anti-tubercular therapy and had been deemed cured by her physician one year ago.

On examination, the patient was febrile, tachycardic, hypotensive, and severely dehydrated. Abdominal examination revealed tenderness in the epigastric and left hypochondriac regions, with voluntary guarding. Laboratory tests showed an elevated total leucocyte count (TLC: 28,000/mm³) and increased inflammatory markers, including C-reactive protein (CRP). Renal function tests (RFTs) and serum bilirubin levels were also marginally elevated, while other laboratory values were unremarkable (Table 1).

**Table 1:** Laboratory values of the patient Inference: leukocytosis (lymphocytosis); borderline elevated serum total bilirubin levels and liver enzymes; elevated CRP levels (acute phase reactant)

Variables	Patient values	Reference values
Total bilirubin	1.4 mg/dl	0.22-1.20 mg/dl
Conjugated bilirubin	0.2 mg/dl	Upto 0.5 mg/dl
Unconjugated bilirubin	1.2 mg/dl	0.1-1.0 mg/dl
Serum glutamic-oxaloacetic transaminase (SGOT)	74 U/L	8-48 U/L
Serum glutamic pyruvic transaminase (SGPT)	80 U/L	7-55 U/L
Alkaline phosphatase	135 U/L	40-129 U/L
C-reactive protein (CRP)	50 mg/L	Upto 5.0 mg/L
Urea	80 mg/dl	17-49 mg/dl
Serum creatinine	1.4 mg/dl	0.6-1.3 mg/dl
Haemoglobin (Hb)	12.2 g/dl	13.2-16.6 mg/dl
Total leukocyte count (TLC)	12800/uL	4000-10000/u l

**Figure 1:**
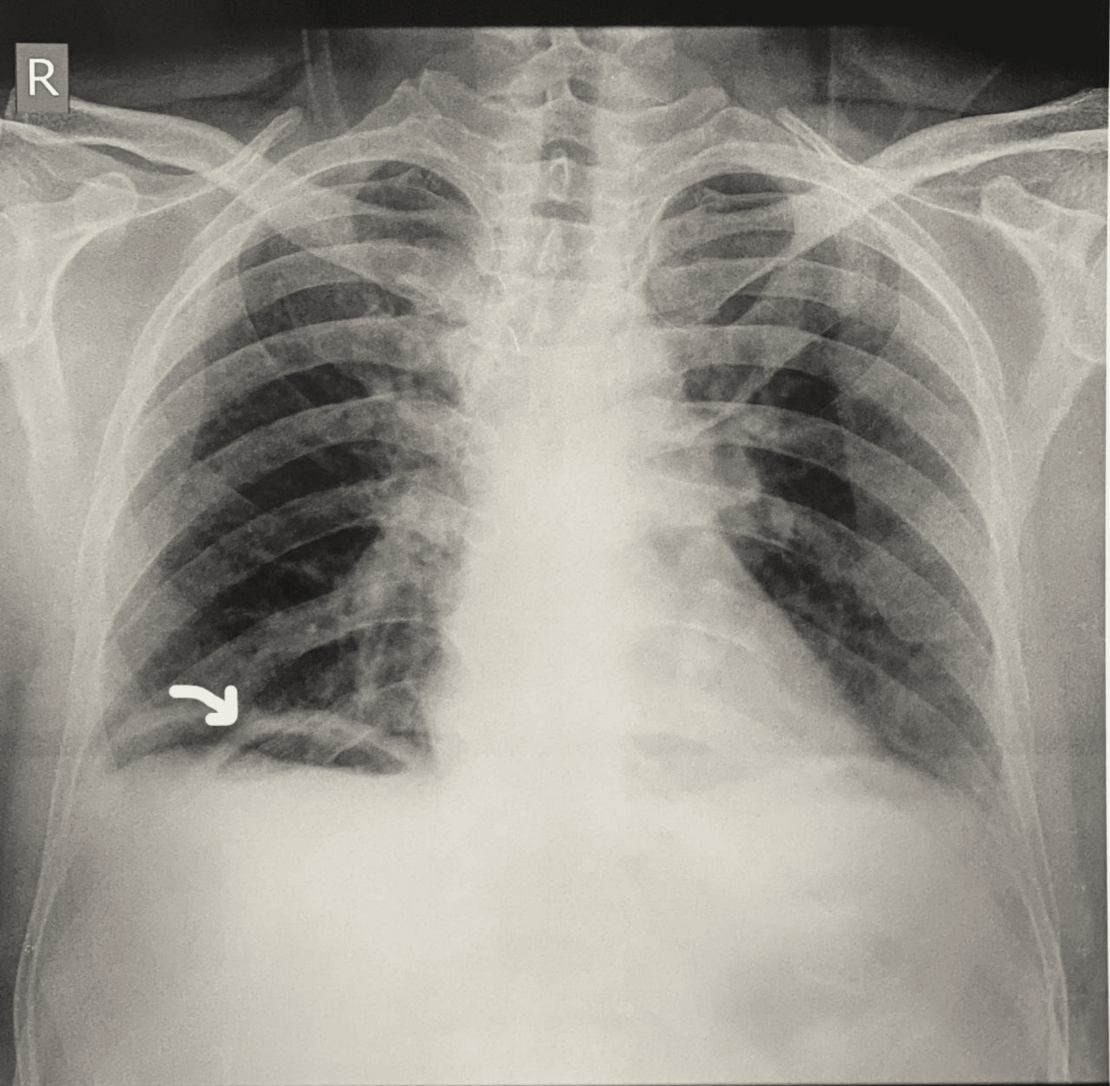
Chest X-ray (preoperative) Chest X-ray showing free air under the right dome of the diaphragm (white arrow)

Initial management included resuscitation in the surgical ICU (SICU), insertion of a nasogastric (NG) tube, and initiation of broad-spectrum antibiotics. An erect abdominal X-ray and chest X-ray revealed gas under the right dome of the diaphragm and multiple air-fluid levels, indicative of pneumoperitoneum and peritonitis (Figure 1). Given the clinical severity and imaging findings, the decision for surgical intervention was made after explaining the risks to the patient’s relatives.

**Figure 2:**
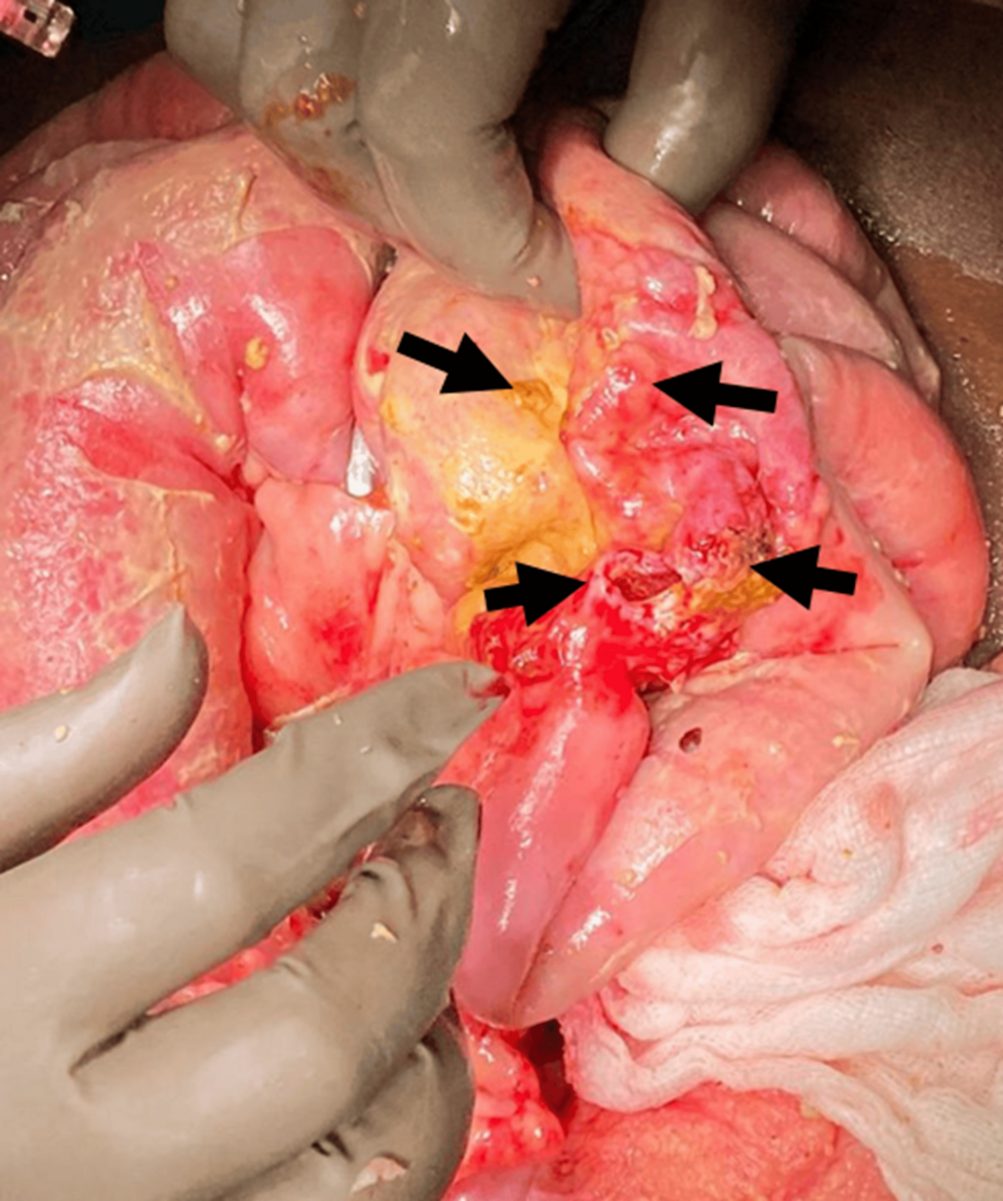
Intraoperative image Multiple perforations in the mid-jejunum over a segment of 10 cm and dilation of the jejunum proximal to the stricture up to 6 cm (wall-to-wall diameter)

**Figure 3:**
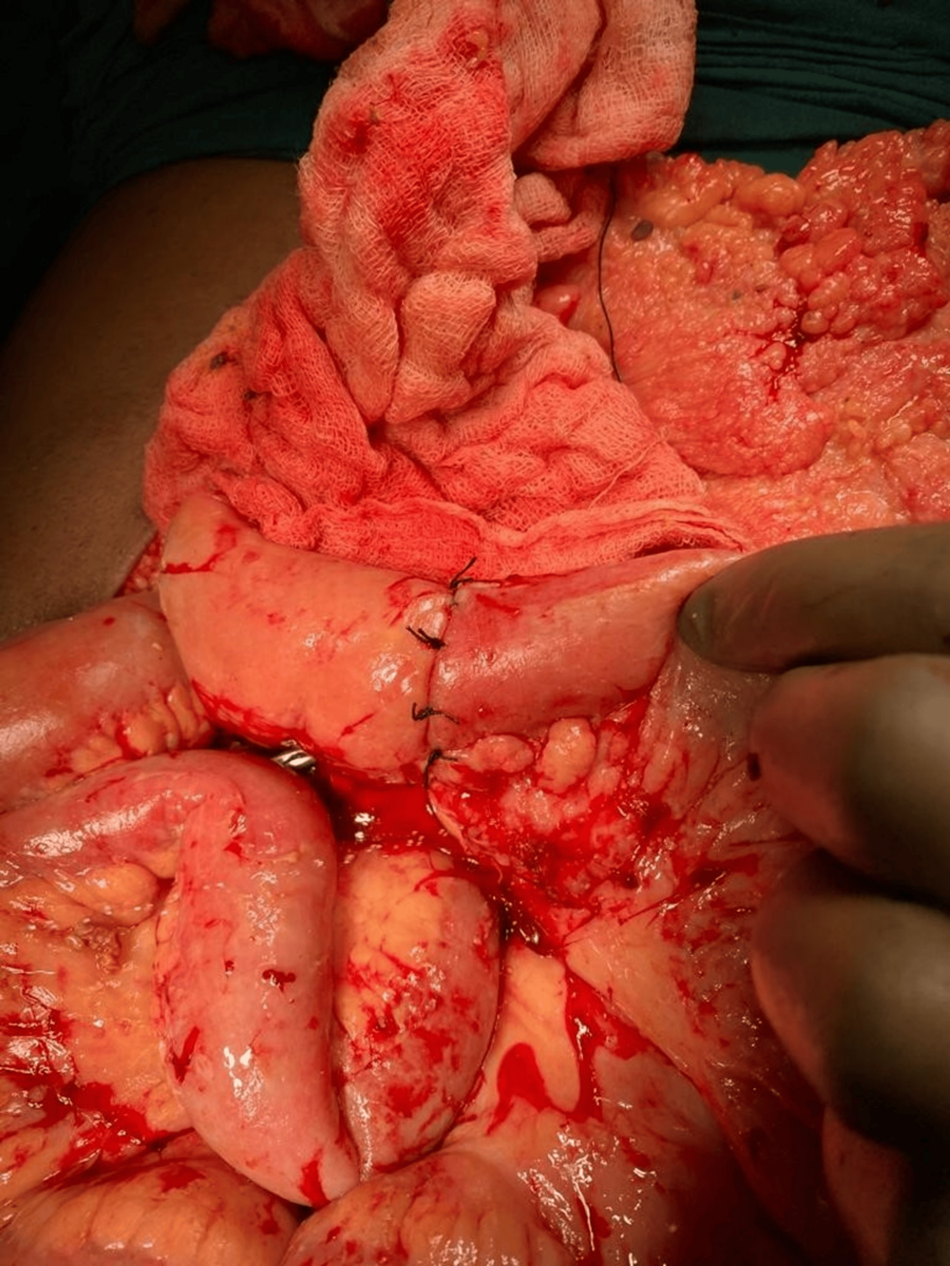
Intraoperative photograph showing intestinal perforation Resection and anastomosis being performed (jejunoileal end-to-end anastomosis in four layers)

During exploration, approximately 2000 ml of fecal-stained fluid was aspirated. Four perforations were identified in a 10 cm segment of the mid-jejunum, with a stricture distal to the perforations causing proximal jejunal dilatation of about 10 cm (Figure 2). The diseased segment was resected, and bowel continuity was restored with an end-to-end jejunoileal anastomosis (Figure 3). The surgery was completed without any complications, and the patient experienced a smooth postoperative recovery.

**Figure 4:**
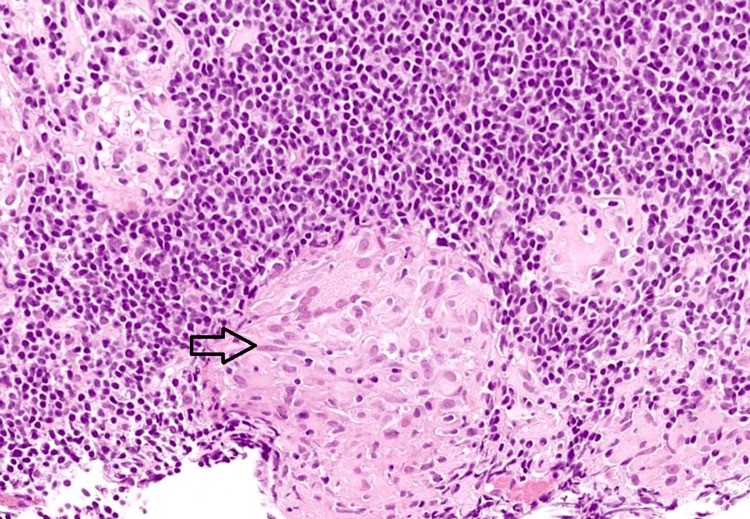
Histopathology image of intestinal mucosa from the perforation margin The image shows caseating granuloma (black arrow) with lymphocytic proliferation

Histopathological examination confirmed TB as the causative agent of the jejunal perforations (Figure 4). The patient was discharged on the 14th postoperative day with a nine-month course of anti-tubercular medications. At her one-month follow-up visit, she was symptom-free, and her follow-up laboratory values showed normalization of previously elevated parameters. Six months post-surgery, the patient did not show any evidence of intestinal obstruction secondary to either stricture formation at the anastomotic site or the development of a new lesion. She completed her anti-tubercular treatment (Category I - Revised National Tuberculosis Control Programme - RNTCP) and did not have any significant side effects. A regular follow-up is still maintained to monitor for the possible sequelae of intestinal TB.

## Discussion

TB remains a significant global health issue, particularly in countries with high prevalence rates such as India, which accounts for a substantial proportion of the cases worldwide [6]. The World Health Organization (WHO) estimates approximately 9.4 million new TB cases annually, with 1.98 million originating in India alone, and the disease is responsible for about 500,000 deaths per year in the country. India and a few other nations hold over 50% of the global TB burden, emphasizing the critical need for effective management and early detection strategies to combat this scourge [7].

In the context of extrapulmonary TB, gastrointestinal involvement is notable, with the ileocecal area being the most commonly affected. Gastrointestinal TB, although not the most frequent extrapulmonary manifestation, represents a significant diagnostic challenge due to its symptom overlap with other abdominal conditions [8]. The diagnosis is often delayed because gastrointestinal TB can present in ways that mimic other disorders, and pulmonary TB is present in fewer than 25% of gastrointestinal TB cases [9]. The tubercular bacillus may reach the gastrointestinal tract through several routes, including ingestion of infected sputum, hematogenous spread, or less commonly, direct spread from adjacent infected tissues.

Strictures are a common complication of gastrointestinal TB, arising from reactionary fibrosis, cicatricial healing of ulcers, and occlusive vascular changes leading to ischemia [10]. Recent advances in molecular diagnostics, such as polymerase chain reaction (PCR)-based tests, have improved sensitivity and may aid in more accurate and earlier diagnosis [4]. Conventional anti-tubercular therapy, typically administered for at least six months, remains the cornerstone of treatment. The management of intestinal obstruction due to strictures remains controversial, with options ranging from surgical intervention to a trial of anti-tubercular medication. Surgery is generally considered only if there is inadequate response to medication [11].

The effectiveness of the current anti-tubercular regimen is well-established, but its impact on intestinal lesions, particularly those with stenosis, can be complex. There is evidence suggesting that treatment may contribute to further intestinal constriction and fibrosis, making surgical intervention necessary in some cases [12]. Despite the efficacy of medications such as rifampicin, which is associated with increased scarring and blockage, timely surgical intervention can prevent severe complications like perforation, which carries significant risk [13,14]. The choice of surgical approach depends on various factors including disease severity, gut condition, and patient health.

Effective management of TB, especially in patients with gastrointestinal involvement, requires a multi-faceted approach that includes early detection, appropriate use of diagnostic tools, and a combination of medical and surgical interventions when necessary. Increased awareness and health education are crucial both in countries with high TB prevalence and in regions with significant migrant populations. Clinicians must remain vigilant for complications and be prepared to adjust treatment strategies to address the disease's diverse manifestations and potential complications effectively.

## Conclusions

This case highlights the critical importance of considering TB in the differential diagnosis of small bowel perforations, particularly in regions with high TB prevalence. Prompt surgical intervention and appropriate anti-tubercular therapy were essential for the successful management of our patient. This case underscores the need for heightened clinical awareness and a multidisciplinary approach to effectively diagnose and treat gastrointestinal TB, ultimately improving patient outcomes.
